# Beyond Antioxidant Effects: Nature-Based Templates Unveil New Strategies for Neurodegenerative Diseases

**DOI:** 10.3390/antiox10030367

**Published:** 2021-02-28

**Authors:** Andrea Bacci, Massimiliano Runfola, Simona Sestito, Simona Rapposelli

**Affiliations:** 1 Department of Pharmacy, University of Pisa, Via Bonanno 6, 56126 Pisa, Italy; andrea.bacci@phd.unipi.it (A.B.); massimiliano.runfola@farm.unipi.it (M.R.); 2 Department of Chemistry and Pharmacy, University of Sassari, Via Vienna 2, 07100 Sassari, Italy; ssestito@uniss.it

**Keywords:** antioxidant, natural products, neurodegenerative diseases, oxidative stress, multitarget, Alzheimer’s disease, drug discovery

## Abstract

The complex network of malfunctioning pathways occurring in the pathogenesis of neurodegenerative diseases (NDDs) represents a huge hurdle in the development of new effective drugs to be used in therapy. In this context, redox reactions act as crucial regulators in the maintenance of neuronal microenvironment homeostasis. Particularly, their imbalance results in the severe compromising of organism’s natural defense systems and subsequently, in the instauration of deleterious OS, that plays a fundamental role in the insurgence and progress of NDDs. Despite the huge efforts in drug discovery programs, the identification process of new therapeutic agents able to counteract the relentless progress of neurodegenerative processes has produced low or no effective therapies. Consequently, a paradigm-shift in the drug discovery approach for these diseases is gradually occurring, paving the way for innovative therapeutical approaches, such as polypharmacology. The aim of this review is to provide an overview of the main pharmacological features of most promising nature-based scaffolds for a possible application in drug discovery, especially for NDDs, highlighting their multifaceted effects against OS and neuronal disorders.

## 1. Introduction

Neurodegenerative diseases (NDDs) consist of a broad class of pathological conditions characterized by a progressive and irreversible degeneration of the nervous tissue, mainly occurring in the elderly. As life expectancy increases, the lack of efficient treatments able to halt or slow down neurodegenerative processes is turning NDDs into a huge socio-economic challenge for healthcare systems. At the current trends, the global population of those over 60 years of age is forecasted to exceed 2 billion by 2050, with a consequent increase of NDDs, such as Alzheimer’s (AD), Parkinson (PD), Huntington’s Disease (HD), and amyotrophic lateral sclerosis (ALS) [1]. Despite the vast heterogeneity of NDDs clinical phenotypes, high levels of oxidative stress (OS) have been identified as a prominent hallmark in their pathogenesis [2]. Beside lipid peroxidation products (e.g., acrolein) that have been found in high levels in patients affected by NDDs, high levels of other OS biomarkers such as malondialdehyde (MDA) and 4-hydroxynonenal (HME) have been detected in PD, while high levels of 8-hydroxy-2′-deoxyguanosine (8-OHdG) and nitrotyrosine characterize HD; also, ALS tissues have shown high concentrations of of dihydropyrimidinase-related protein 2 (DRP-2), heat shock protein 70, and α-enolase [3]. 

OS is well-known to play a key role in premature ageing, contributing to the progressive loss of tissue and organ function. At the same time, OS is implicated in several age-related conditions, including cancer, cardiovascular, neurodegenerative, and inflammatory diseases. This phenomenon rises from an imbalance between oxidative and reductive processes occurring during physiological metabolism [4,5]. In pathological conditions reactive oxygen species (ROS) could disrupt cells’ membranes and deeply damage cellular components through a series of peroxidative reactions (e.g., lipid peroxidation) [6]. Moreover, ROS can induce oxidative modifications of proteins and DNA damage. The central nervous system (CNS) is particularly vulnerable to these events due to the large amount of oxygen required for neuronal metabolism and the high concentration of lipids prone to peroxidation that constitute neuronal membranes. Much evidence has also associated cellular ageing to harmful oxidative events induced by metabolic waste products and their accumulation, along with a progressive inefficiency of physiological defence and repair systems (e.g., autophagy) [7]. Furthermore, ageing is associated with gene mutations that lead to cellular malfunctions such as mitochondrial and cellular genome variations, reduced protein biosynthesis, and lipid accumulation [8]. Interestingly, ROS have a prominent role in all these processes. Indeed, further studies performed in animal models reveal the existence of strong interconnection between ROS and metallostasis alterations, protein aggregation, and mitochondrial failures that characterize NDDs. Over the years, the research of new anti-ageing and nature-based agents able to counteract cellular senescence and neurodegeneration, has focused to the identification of small molecules with antioxidant activity; unfortunately, their therapeutic potential seems to be limited, showing that targeting ROS accumulation did not lead to the expected results [9]. Additionally, severe side-effects and poor blood brain barrier permeability are few of the main limits that have undermined their translatability in human model and consequently, their potential therapeutic application [10]. Hence, a novel and paradigm-shifting view on NDDs seems necessary to achieve better treatments of these conditions. Particularly, identification of treatments targeting several pathogenic pathways—i.e., polypharmacology—has emerged as a new pharmaceutical strategy that could propel beneficial effects counteracting the multi-faceted impairment of NDDs. A huge contribute in designing and developing polypharmacological compounds may come from nature. Indeed, a large number of activities—such as antioxidant, chelating, and anti-inflammatory—have been associated to natural compounds [11]. Furthermore, recent studies have proved that many natural compounds are able to regulate autophagy and proteasomal degradation pathways and to curb protein misfolding [12,13]. On this basis, combination of the wide-range benefits provided by natural compounds could represent an attractive therapeutic strategy in order to obtain an effective multi-functional treatment in preventing neurodegeneration. The aim of this review is to provide an overview of the main pharmacological features of most promising natural antioxidants with a potential therapeutic application in the drug discovery process against NDDs.

## 2. Crucial Role of ROS in Physiological and Pathological Mechanisms

High rates of oxygen metabolism, along with the abundant distribution of redox-active metals and polyunsaturated fatty acids, are some critical points in the neuronal microenvironment [8]. The two unpaired electrons on the O_2_ have high reactivity to form a group of free radicals called ROS. These species can originate from exogenous sources (irradiations or chemicals), although the main source of endogenous ROS remains the respiratory chain of mitochondria and nicotinamide adenine dinucleotide phosphate (NADPH) oxidase enzyme (NOX). The mitochondrial electron transfer chain (ETC) consists of five complexes able to modulate ROS production [12,13]. Besides NOX enzyme, further enzymatic systems and cellular organelles are involved in ROS production: xanthine oxidases (XO) transfer electrons to O_2_ generating the superoxide O_2_^•−^ and H_2_O_2_; the endoplasmic reticulum (ER) may produce ROS as by-products; and peroxisomes, containing several oxidases enzymes, could increase the release of H_2_O_2_ in the cytosol [14]. Collectively, these mechanisms generate O_2_^•−^ which does not directly interact with the cellular substrates, but has a crucial role in free radicals production involved in maintaining physiological functions, including proliferation, defense against infectious agents, signal transduction, and gene expression [13]. 

Physiologically, low and beneficial ROS levels are preserved through the action of several antioxidant enzymes. For example, superoxide dismutase (SOD) inactivates O_2_^•−^ by conversion into H_2_O_2_, that can be then removed by catalases and glutathione peroxidases (GPX), producing H_2_O and O_2_. Tonal levels of ROS have a wide range of significant effects in physiological cellular signaling and survival mechanisms. For example, ROS can activate mitogen-activated protein kinases (MAPKs) that constitute a crucial pathway in cardiovascular system [15]. Other important pro-survival transcription factors—such as NF-E2-related factor 2 (Nrf2) and nuclear factor-κB (NF-κB)—are influenced by ROS levels [16]. An imbalance between ROS production and antioxidant defenses contributes to the insurgence of OS, concurring to cellular disfunctions, ageing, and neurodegeneration [17]. Indeed, chronic OS is a well-known pathogenic factor with a prominent role in the etiology of several NDDs. Many peculiar cellular disfunctions and pathological phenomena in neurodegenerative tissues are due to ROS accumulation. Additionally, ROS can promote DNA, RNA, and protein oxidation, lipid peroxidation, mitochondrial failure, and protein aggregation. The overwhelming production or the shortage of ROS levels are both deleterious for cellular homeostasis and play an important role in the malfunctioning process of mitochondria, cells and organisms [14,15]. ROS levels could also affect non-cell autonomous effects, which contribute to the neurodegenerative process mainly through an uncontrolled activation of inflammatory status and immune response. Both neurons’ environmental factors, such as increased OS, either endogenous factors—e.g., protein aggregates accumulation—induce an over-activation of the innate immune cells in the CNS, such as microglia and astrocytes. This uncontrolled inflammatory status results in the production of neurotoxic factors that contribute to amplify the disease states and actively participate in the pathological vicious circle [16].

### 2.1. ROS and Mitochondrial Dysfunctions

Under normal conditions, ROS are finely regulated, and their concentration kept under control: only about 2% of oxygen consumption in mitochondria is converted to ROS. Recently, Aon et al. proved that this percentage could fluctuate from 0.25% to 11% when the endogenous antioxidant systems are disabled [17]. Basal levels of ROS initiate and coordinate several pathways finely tuned with cellular demands. For example, ROS are required for physiological regulation in cycle progression and their levels are associated with cell differentiation, migration, and proliferation; immune response; and apoptosis [18,19]. Hereby, oxidative homeostasis appears to be fundamental for normal mitochondrial functions. Mitochondria are considered the energy powerhouse of cells. Indeed, they are the main source of cellular ATP, and play important roles in ion homeostasis, metabolic pathways, apoptosis, and in ROS production and consumption [20]. Increased levels of oxidative damage contribute to metabolic stress and cellular injuries, and the mitochondrial DNA (mDNA) represents a critical target for such reactive species. The mitochondrial genome is located close to the inner membrane where the respiratory machinery produces ROS, making mDNA a primary target of ROS and therefore highly exposed to damage, deletion, and mutation [21]. Once mDNA is damaged, mitochondria can raise OS and enter a vicious cycle that led to an increase of ROS production. This phenomenon is called “ROS-induced ROS release” (RIRR) and results in a boosted superoxide production contributing to metabolic OS, genome instability, and cellular lesions [22]. ROS abundance increases genomic instability; several studies confirmed indeed that a marked ROS accumulation is often associated with an increased rate of DNA mutations in mice [14], rats [15], nematodes [23] and humans [24]. Besides mDNA, mitochondrial membranes are highly exposed to ROS-induced damages. The inner membrane is the site where ETC and phosphorylation happen; under physiological conditions its permeability is tightly regulated and allows exclusively the permeation of small neutral molecules. Redox stress may induce the opening of membrane anion channel (IMAC) and mitochondrial permeability transition pore (mPTP), both responsible for mitochondria swelling, thus leading to the collapse of mitochondrial bioenergetic functions and ultimately to cellular death [25]. In this process, ROS are involved in activating IMAC and mPTP; consequently, mitochondria undergo an increase in ROS production through a positive-feedback mechanism [26]. Moreover, high levels of ROS lead to an uncontrolled peroxidation of lipids and phospholipids that constitute cellular membrane, thus altering its biophysical properties, such as fluidity and permeability. These biophysical ROS-induced alterations can also impair the activity of various transporters and respiratory proteins within the inner membrane [27]. Oxidation of thiol moieties of the adenine nucleotide translocator located on mPTP may also promote variations in mitochondrial permeability [28]. Finally, many other enzymes (i.e., nicotinamide adenine dinucleotide dehydrogenase and succinate dehydrogenase) are vulnerable to oxidative alterations of their iron-sulfur centers, and their impairment could induce malfunctions of the ETC [29]. Alterations in membrane permeability promote a change in mitochondrial potential linked to disruption of Ca^2+^ homeostasis and the consequent overproduction of O_2_^•−^ [30]. The increased Ca^2+^ levels could also promote osmotic swelling and the break of mitochondrial membrane, mPTP’s opening, and thus apoptosis [31]. Altogether, high levels of ROS impair cellular homeostasis, leading to harmful effects on biomolecules that ultimately contribute to cellular senescence and, more importantly, to the pathogenesis and progression of neurodegenerative diseases [32,33,34,35].

### 2.2. Metal Accumulation, ROS Production, and Protein Misfolding

Along with ROS, metals play a significant role in tuning enzymes activity. Alteration in metal homeostasis, namely metallostasis, is an additional key factor in OS insurgence, leading to cellular ageing and NDDs. For decades metals accumulation has been studied as a prominent mechanism in neuro- and cardiotoxicity, generation of free radicals, lipid peroxidation, and protein aggregation [36,37]. Essential biometals—like iron, copper, zinc, manganese, calcium, magnesium—and non-essential metals—like aluminium, lead, and mercury—seem to play a crucial role in the development and progression of neurodegenerative diseases [38,39]. Indeed, multi-valence metals can support ROS cascade determining an enhanced generation of toxic radicals. For example, iron, copper, aluminium, and zinc can take part in Fenton and Haber–Weiss reactions: the first is defined as the reaction between Fe^2+^ and H_2_O_2_ generating Fe^3+^ and hydroxyl radical, then converted to O_2_^•−^; the latter forms hydroxyl radical and hydroxyl anion from reaction of O_2_^•−^ with H_2_O_2_ [22,40,41]. Therefore, an accumulation of metals can generate and support ROS overproduction, overwhelming the endogenous antioxidant defence systems and contributing to OS insurgence [42]. Additionally, metals can exert direct damages to biostructures, such as proteins, interacting with some specific structural regions and destabilizing their native conformation. Multivalent metals can bridge together charged aminoacidic residues of proteins, especially when these residues are prominently exposed like in compromised or misfolded proteins [43]. The correlation between metals and protein aggregation has been widely studied and many evidence suggest that metals accelerate the cytotoxicity of proteins deposition [44,45]. Accordingly, metals accumulation in brain and altered metallostasis are both hallmarks of neurodegenerative disorders. However, it remains unclear if metals accumulation is a leading cause in the onset of NDDs or the result of previous dysfunctions [46]. Nevertheless, among the strategies pursued to limit OS injury and the consequent neurodegeneration, metal chelation appears as an attractive tool to counteract metal-induced cellular damages [47]. To this end, chelating agents have been widely studied and their therapeutic role in CNS disorders and cellular aging continuously investigated [48].

### 2.3. OS and Protein Misfolding/Accumulation

Another prominent hallmark of many age-related diseases is the impairment of protein homeostasis. Proteostasis dysregulation leads to an accumulation of toxic misfolded and abnormal protein aggregates associated with common NDDs such as AD, PD, ALS, and frontotemporal dementia (FTD). Proteostasis is maintained by proteolytic machineries and their regulators; all of them operate through an extensive monitoring network that makes use of degradation pathways to clear toxic misfolded proteins. During ageing, the efficiency of this network declines over time, leading to a progressive proteostasis imbalance. There are strong evidence indicating that two of these protein degradation systems, the ubiquitin–proteasome system (UPS) and the autophagy–lysosome pathway (ALP) become progressively compromised with ageing [49]. Actually, the impairment of these degradation systems promotes an accumulation of misfolded and abnormal proteins, including amyloid-beta (Aβ) in AD, huntingtin (HTT) in HD, superoxide dismutase-1 (SOD1) and TAR DNA-binding protein 43 (TDP-43) in ALS, and α-synuclein in PD [50]. Proteostasis impairment mostly occurs through irreversible oxidative modifications of different proteins, mainly induced by OS [51]. Beyond the existence of a cross-talk between ALP and UPS, recent biochemical studies suggest a direct interplay of the proteolytic machinery with OS, thus contributing to the aetiology and the progression of neuropathogenic cellular conditions [52]. As a matter of fact, UPS plays a key role in physiological protein turnover, recognizing and degrading insoluble, damaged, and oxidized proteins, preventing their cellular accumulation. OS could aggravate the ageing-related decline of misfolded proteins clearance, entering a self-propagating cycle of ROS-induced protein aggregation and malfunctioning of the proteostasis network [53]. Likewise, autophagy has been proposed as anti-ageing mechanism as it sustains neuronal survival promoting clearance of protein aggregates [54]. Additionally, ALP contributes to proteostasis maintenance driving the degradation of cytotoxic aggregates and preventing their cellular accumulation. Oxidative damage could be reduced by autophagic machinery by removal of unnecessary or damaged organelles, or by limiting the excessive ROS activation in response to neuronal damage [55]. Protein aggregates and ROS-induced dysfunctional mitochondria can propagate damage in surrounding cells, causing further protein aggregations, and spreading oxidative cellular stress, ultimately leading to neurodegenerative processes. Indeed, scavenging of free radicals by antioxidant systems cannot completely control peroxidation and protein aggregation, indicating that autophagy has an essential and effective role in neuronal survival and OS decline [56]. Recent studies revealed that OS can also impair autophagy via direct oxidation of catalytic thiols on several proteins involved in the autophagic cascade, such as autophagy-related (ATG) proteins ATG3 and ATG7 [57]. Therefore, protein accumulation significantly contributes to OS and, on its turn, exacerbates the age-related production and aggregation of misfolded proteins. This synergistic correlation affects both etiology and progression of NDDs, representing either a potential biomarker for diagnosis and a valuable target for the treatment of OS-related proteinopathies [58]. The most relevant physiological and pathological roles addressed to ROS are summarized in Table 1.

## 3. Nature-Based Compounds against Cellular Aging and Neurodegeneration

Historically, several nature-based compounds from dietary intake—including polyphenols, terpenes, and organosulfur compounds—have shown beneficial effects against OS, cellular ageing, and neurodegeneration mainly associated with their antioxidant activities [59]. Through the years, research in this field has focused on elucidating and exploring the pharmacological profile of natural products against OS and their therapeutic application for NDDs [60]. Beside antioxidant properties, phytonutrients such as quercetin, resveratrol, curcumin, and genistein can propel beneficial effects through activation of different pathways, including autophagy [61,62]. Due to their prominent polypharmacology, natural compounds have been widely employed in drug discovery and their chemical scaffolds frequently used as starting points in the design of new therapeutic agents for several pathologies [63]. More recently, and with the rise of nutraceutics and functional food, a massive number of studies identified polyphenols as potential anti-ageing and neuroprotective molecules that could find application for preventing and/or treating chronic diseases [64,65]. In the next paragraphs, we will discuss promising and attractive properties of selected nature-based scaffolds for a possible application in drug discovery, especially for NDDs, highlighting their multifaceted effects against OS and neuronal disorders.

### 3.1. Polyamines: Spermidine and Spermine

**Figure 1:**
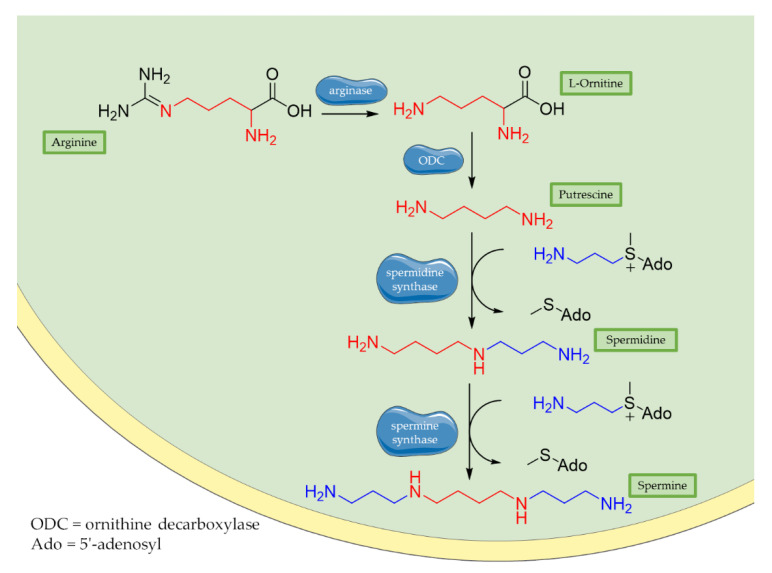
Biosynthesis of polyamines. Briefly, arginase operates the removal of guanidino group from essential amino acid arginine, producing L-ornithine. Then, decarboxylation of L-ornithine mediated by ODC enzyme generates putrescine that is converted to spermidine by spermidine synthase that adds an aminopropyl group to the molecule. Finally, addition of a second aminopropyl moiety to spermidine generates spermine.

Historically, polyamines were first discovered by van Leeuwenhoek in 1678 as crystalline substances in seminal plasma, but their structures were characterized only in the 20th century by Rosenheim [66]. Different concentrations of polyamines have been found in fruits, vegetables, and food of animal origin [67]. Spermidine, spermine, and their common diamine precursor putrescine are natural amines distributed widely in all cells, and their biosynthesis, degradation, and membrane transport are finely tuned in mammalians. The first step in polyamines biosynthesis requires the conversion of arginine to L-ornithine catalysed by the ureo-hydrolytic enzyme arginase. Putrescine is then obtained from ornithine through decarboxylation catalyzed by ornithine decarboxylase enzyme (ODC). Finally, spermidine and spermine synthases catalyse the biosynthesis of spermidine and spermine, respectively, adding an aminopropyl group to putrescine or to spermidine (Figure 1) [68]. 

Polyamines exert a wide range of beneficial effects against cellular aging and OS. Endogenous levels of spermidine decrease with age but remain stable in those older than 90 years of age, suggesting that it may contribute in longevity and anti-aging protection [69]. Spermine and spermidine are well-known for their activity as direct ROS scavengers providing DNA protection from OS [70,71]. Both in vitro and in vivo studies demonstrate that the antioxidant and anti-inflammatory properties of spermidine are associated with decremental cellular levels of nitric oxide synthase (NOS), prostaglandins and cytokines.: such as NOS inducible isoform (iNOS), prostaglandine E2 (PGE2), and pro-inflammatory cytokines, like interleukin-1β (IL-1β) and tumor necrosis factor-α (TNF-α). In addition, spermidine inactivates NF-κB in macrophages influencing pro-inflammatory genes expression [72]. Autophagy has been identified as one of the main mechanisms responsible for anti-ageing effects of spermidine, even though more pathways are affected by this molecule such as cell proliferation and differentiation, lipid metabolism and inflammation [73]. The polycationic nature of polyamines drives their capacity to interact with negatively charged molecules, such as DNA, RNA, proteins, and lipids. For this reason, they are involved in various processes, including DNA stability/repairment and proteins and nucleic acids synthesis [74,75]. Spermidine is also correlated with both hyper- and hypo-acetylation of proteins involved in the autophagic process [76,77]. Spermidine competitively inhibits the acetyltransferase EP300, an autophagy inhibitor that directly acetylates and blocks several ATG complexes and microtubule-associated protein 1A/1B-light chain 3 (LC3), providing a stimulation of autophagic flux in mammalian cells [78]. Autophagy promotion induced by spermidine is also mediated by its inhibitory effect on histone acetyltransferase enzyme P/CAF leading to histone H3 hypoacetylation. Histone acetylation and DNA interaction seem to be involved in the synergistic mechanism by which spermidine, and likely other polyamines, promote autophagy and influence chromatin structure [79]. Another study showed that spermidine also prolongs lifespan and reduces OS in mice models of hepatocarcinoma. These effects are due to the increased acetylation of microtubule-associated protein 1S gene (MAP1S) and consequently autophagy promotion [80]. Moreover, spermine showed a good potential towards clearance of misfolded proteins in prion infected cell cultures [81]. Additionally, it increases acetylation of microtubules, thus enhancing the retrograde transport of autophagic vesicles to lysosomes. Spermine offers also an epigenetic control associated with DNA-methylation: it seems to exert anti-inflammatory protection via DNA methyltransferase (DNMT) activation that regulates methylation of the entire genome as well hypermethylation of Integrin Alpha L (ITGAL) gene. Hypermethylation of ITGAL is correlated with the suppression of inflammatory status [82]. In vivo studies revealed that spermidine exerts a neuroprotective effect in brain injured mice showing reduction of pro-inflammatory cytokines and traumatic brain injury biomarkers. This neuroprotection is given also by a spermidine-driven autophagic activation. In brain samples, after spermidine treatment, Beclin-1 and LC3 markers are upregulated, thus confirming an increase of autophagic activity also in vivo [83]. Finally, polyamines proved to ameliorate cognitive functions. Intrahippocampal co-administration of spermidine and arcaine (an antagonist of polyamine binding site at NMDA receptor) in mice exerts a modulatory effect on memory, presumably by activating NMDA receptors [84]. 

Despite this plethora of effects and their chemical appeal, polyamines are quietly unexplored as therapeutic tools in designing new multi-target drug candidates for NDDs treatments. One of the first polyamine-based papers on this topic dealt with merging polyamines with the 1-aminoindan scaffold, in order to improve the lipophilicity of the new molecules and then promote the transport at the CNS through biological membranes. Among the new compounds synthesised by Gilad et al., compound 2711 (1, Table 2) compared with natural polyamines, showed to enhance the neuroprotective effect on damaged brain tissue, proving either a potent neuroprotective effect in in vitro/in vivo experimental models of neurotrauma and a good capability to reach the CNS [85]. Accordingly, in 2010 Melchiorre and collaborators reported memoquin as a new compound synthesized following the multi-target ligand design (MTLD) approach [86]. Memoquin (2, Table 2) was obtained by the combination of the 1,4-benzoquinone scaffold (a radical scavenger) with the polyamine structure of caproctamine, an acetylcholinesterase (AChE) inhibitor and muscarinic M_2_ receptor antagonist. Both in vitro and in vivo assays demonstrated that treatment with memoquin can affect a wide range of pathogenic mechanisms involved in AD, including Aβ aggregation, tau hyperphosphorylation, OS, and AChE and BACE-1 activities [87]. The alkyl-2-methoxy-benzyl fragment of memoquin, which seems to play a key role in AChE inhibitory activity, was also combined with ferulic acid (FA) using polyamine linkers (3, Table 2). New molecules originated with this approach showed a pleiotropic activity with an improved antioxidant profile [88]. Similarly, Zhang and colleagues merged antioxidant isoflavonoid scaffold of genistein with various polyamines, to identify new multifunctional anti-AD agents (4, Table 2). New designed agents were able to inhibit AChE and butyrylcholinesterases (BuChE), and showed chelating properties towards Fe^3+^, Cu^2+^, and Zn^2+^, without inducing cytotoxicity in vitro [89]. Again, Simoni et al. conjugated 3,5-dibenzylidenepiperidin-4-one bioactive motives with spermine to target amyloid aggregation as a promising strategy in AD treatment (5, Table 2). Dicatecholic derivatives showed good inhibition of Aβ_42_ aggregation, even though they did not show any antioxidant properties in the same in vitro model [90]. Furthermore, in silico studies revealed an additional key functional role of spermine suggesting that it is directly involved in interactions between Aβ_42_ monomers. The discovery of dicaffeoylspermidine derivatives with antioxidant properties from wolfberry prompted Gao et al. to design dicaffeoylspermidine cyclized derivatives (6, Table 2). These compounds showed significant antioxidant activity in vitro along with an improvement of memory and cognitive functions in fruit flies’ model of senile dementia [91,92]. Taken together, these studies suggest a new and unstudied pharmaceutical potential of polyamines in drug discovery that could be exploited to design new pharmacological agents against NDDs.

### 3.2. Phenolic Acids

For decades, long-term consumption of polyphenols-rich food has been correlated with beneficial effects in human health and protection against cancers, cardiovascular, and neurodegenerative diseases [93]. Polyphenols are a large class of natural compounds characterized by a chemical scaffold with multiple phenolic functionalities and endowed of several biological activities [94,95]. Among these, phenolic acids, have attracted a growing interest in the pharmaceutical field for their strong antioxidant nature and low toxicity [96,97,98]. Their antioxidant activity is mainly provided by the high reactivity of the phenolic moiety able to interact with free radicals which are stabilized by delocalization. This effect induced by polyphenols results in a marked modification of radical-mediated oxidation processes [99]. 

**Figure 2:**
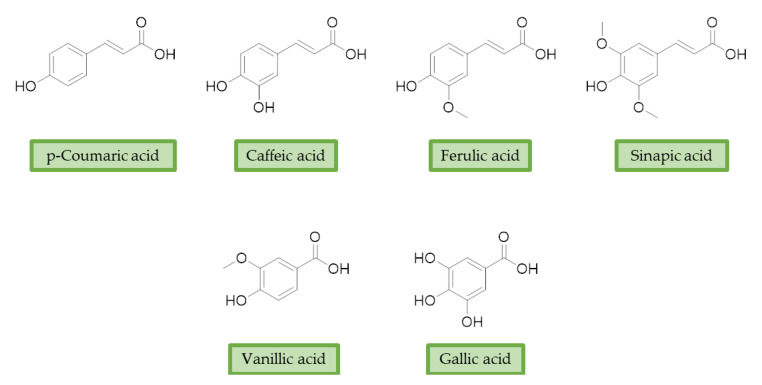
Structures of the main phenolic acids.

Moreover, additional effects have been described in the last years for phenolic acids, like ferulic acid (FA), gallic acid (GA), and caffeic acid (CA) (Figure 2). For example, FA has shown marked neuroprotective effects in a mouse model of cerebral ischemia/reperfusion-induced injury, associated with an increase in SOD and GPH levels, and a consequent reduction of ROS, O_2_^•−^, and Ca^2+^ accumulation. Besides the antioxidant effect, the neuroprotective effect has been also correlated with the downregulation of toll-like receptor 4 (TRL4) and myeloid differentiation primary response 88 (MyD88), both involved in the activation of intracellular NF-κB signalling pathway [100]. Similarly, GA showed a protective role in OS-induced dopaminergic cell lines through two different mechanisms: activation of antioxidant enzymes (i.e., SOD, CAT, GPx, and GR) and regulation of AKT/Keap-1/Nrf2 defence pathway [101]. Polypharmacological profile of phenolic acids is also supported by several studies highlighting their metal chelating properties [102,103], and their ability to positively affect protein aggregation by directly interacting with proteins like Aβ and α-synuclein [104,105]. Additionally, phenolic acids may provide a protective effect regulating autophagy activation. Recent in vitro studies showed that CA is capable of restoring heat shock protein 27 (Hsp27), B-cell lymphoma 2 gene (Bcl-2), and sirtuin 1 (SIRT1) expression, upregulating autophagy, and decreasing mitochondrial ROS by enhancing expression of antioxidant proteins such as GSH, catalase, O-1, NQO-1, and SOD [106]. FA showed protection against vascular dementia by reversing OS state, upregulating LC3-II, and inducing mitophagy in vitro [107,108]. Generally, collected data on phenolic acids underline their cell-protective polypharmacology, suggesting phenolic acids as therapeutic tools for several disorders. 

In order to identify new classes of drugs with enhanced neuroprotective effect and following the well-known multi-target designed ligands (MTDL) approach, a growing number of studies report the combination of phenolic acid scaffold with additional pharmacophoric moieties. Particularly, tacrine and its analogues have been widely employed to this end. Tacrine is a well-known cholinesterase inhibitor, the first AChEI approved by FDA for AD. Besides its primary activity, it also induces OS, due to its capability to promote ROS production and glutathione depletion [109]. A tacrine–ferulic acid hybrid namely T6FA (7, Table 3) was evaluated on Aβ-induced cell death in vitro and in in vivo mice model of AD. Results showed that T6FA enhances cognitive impairment, increasing SOD activity, and limiting AChE activity [110]. In 2018, Zhu et al. designed novel tacrine-ferulic acid hybrids protecting the FA’s free phenolic group with different benzyl moieties. Among these, compound bearing the 3,4-dimethyl benzyl scaffold (8, Table 3) showed an interesting pharmacological profile, inhibiting both AChE and BuChE with an EC_50_ of 37.02 and 101.4 nM, respectively. Moreover, it was able to inhibit Aβ_1–42_ self-induced aggregation in vitro and to improve cognitive impairment in mice model of AD [111]. Similarly, other AChE inhibitors (e.g., the marketed drugs donepezil and rivastigmine), neurotransmitters, or natural products have been combined with FA, CA, and cinnamic acid with the aim of developing new therapeutic tools against AD. Table 3 summarizes some examples of this approach, including effects described within the corresponding references: serotonin (9), aromatic amides and esters (10), donepezil and N,N-dibenzyl(N-methyl)amine (11 and 12), anilides (13), rivastigmine (14), and diallyl sulfide (15) [112,113,114,115,116,117]. Interestingly, most of the compounds synthesised showed a reduction of Aβ-aggregation, mainly imputable to a direct inhibition of the aggregative process itself or to an indirect effect linked to AChE-inhibition. Coherently, novel ferulic derivatives obtained from combination previously synthetized multitarget ligands pharmacophoric groups showed promising results (16, Table 3) [118]. Particularly, TM10 exhibited excellent BuChE inhibitory activity (IC_50_ = 8.9 nM) and high selectivity compared with AChE (IC_50_ = 12.1 μM), along with good antioxidant activity. Moreover, Sang et al. showed that TM10 inhibits Aβ_1-42_ aggregation and promotes disaggregation of Aβ_1-42_ fibrils and induces autophagy [118]. All together the positive results obtained following the MTDL approach confirm that the combination of AChEI with phenolic acids could exert neuroprotective effects by interacting with several pathways such as OS, protein-aggregation and autophagy, all aspects which results to be compromised in several NDDs. In particular, FA has been widely employed in multitarget ligand design because of its polypharmacology and low toxicity even if it still presents some drawbacks like low bioavailability and poor BBB permeability. In 2020, Tripathi et al. synthesized a new series of compounds obtained by the combination of FA with 1,3,4-oxadiazole ring in order to improve the interaction of new synthesised molecules with the active pocket of target enzymes (17, Table 3). All compounds proved a remarkable inhibition of AChE, BuChE, and BACE-1. Moreover, selected compounds were able to reduce Aβ aggregation, exert neuroprotective effects on Aβ-induced damage SH-SY5Y cells and exhibit appreciable BBB permeability [119].

### 3.3. Urolithins

**Figure 3:**
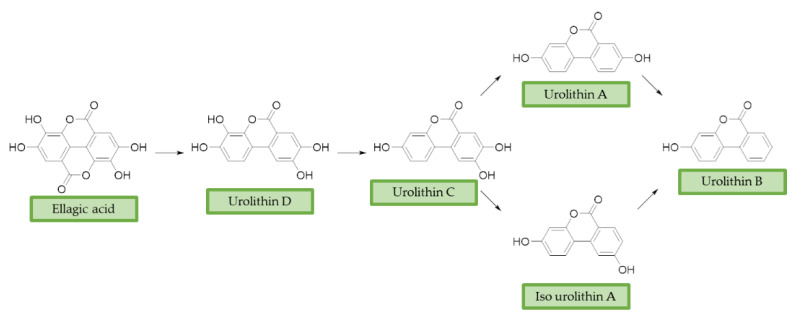
Ellagic acid (EA) and its urolithins metabolites after gut microbiota metabolism. Briefly, after intestinal uptake the hydrolysis and subsequent decarboxylation of EA’s lactone generates urolithin D (UD), that provides UC losing a hydroxyl group. Then, the removal of a phenolic group in 9 or 8 position affords UA or iso-UA, respectively. Finally, UB is produced by removal of the phenolic group in 8 or 9.

Ellagitannins (ET) represent another class of polyphenols, mainly found in strawberries, walnuts, and pomegranates, with prominent beneficial effects on human health [120]. After dietary intake, ET are slightly absorbed and metabolized by the gut microbiota of humans and other animals [121,122]. First, they are converted into ellagic acid (EA) that retains poor bioavailability, then metabolism in the lower gastrointestinal tract convert them in urolithins. Urolithin A (UA), urolithin B (UB), urolithin C (UC), isourolithin A (iso-UA), and their corresponding phase II conjugate derivatives represent the main metabolites found in tissues and plasma [123]. (Figure 3)

EA pharmacological activities and molecular mechanism are still under investigation, but data collected until now are particularly promising. Interestingly, most of beneficial effects associated with EA are attributed to its active microbial metabolites: urolithins are much better absorbed and have been proposed to be responsible of beneficial effects of ET-rich foods [124]. Coherently, over the last decade, researchers’ interests shifted towards urolithins in order to understand their physiological effects and to explore the mechanisms involved. Structurally, urolithins are dibenzopyran-6-one compounds with various hydroxyl groups substitutions, and UA, the 3,8-dihydroxy derivative, appears to be the most abundant metabolite produced in human. UA has shown non-genotoxic and ADME safety profile in short- and long-term oral exposure in rats [125]. Additionally, it demonstrated anti-mutagenic properties in *Caenorhabditis elegans* (*C. elegans*) [126]. Pharmacodynamic studies on pomegranate juice consumption demonstrated that UA can reach micromolar concentrations in humans, without displaying any toxic effects [127]. A prominent interest on neuroprotective effects of urolithins arose upon observation of their scavenger activity against ROS. Recent studies showed that they inhibit intracellular ROS production in vitro, without remarkable cytotoxic effects; interestingly, this effect has been correlated with the number of hydroxyl groups [128]. UA showed neuroprotective effects against H_2_O_2_, reducing ROS production, improving mitochondrial activity and reducing ROS-induced lipid peroxidation in murine neuroblastoma cell lines (neuro-2a). Apart from the direct radical scavenging properties, UA seems to exert its antioxidant activity also through the modulation of antioxidant enzymes. UA increased the expression of peroxiredoxins, a family of thiol-dependent peroxidases involved in redox signaling. This correlation may explain the cytoprotection of UA by improving the activity of other antioxidant defence systems, such as SOD, catalase, and glutathione reductase [129]. Moreover, urolithins showed metal-chelating properties linked to the number and position of hydroxyl groups [102,130]. In the last years, urolithins have also earned interest as potential autophagy modulator. Indeed, several studies indicated that UA can promote autophagy in macrophages [131], colorectal cancer cells [132], and microglia human cell lines [133]. Again, UA showed a neuroprotective effect through autophagic activation, repressing ER stress and attenuating neuronal injuries in mice [134]. By contrast, UB cytoprotective effects has been associated with the modulation of the uncanonical p62/Keap1/Nrf2 pathway, resulting in increased levels of downstream antioxidant enzymes [135]. Despite the wide range of effects against OS and the promising results obtained from studies on biotransformation, metabolism, and physiological effects, the use of urolithins as chemical scaffolds for drug design in the field of NDDs is still relatively unexplored. In 2014, Gulcan et al., combined modified 6H-benzo[c]chromen-6-one core of urolithin with rivastigmine (18, Table 4) and donepezil-like scaffolds (19, Table 4), obtaining a small library of derivatives with promising polypharmacological activity. Selected compounds showed micromolar and sub-micromolar IC_50_ against AChE and BuChE and exhibited comparable activity with donepezil and rivastigmine, in a scopolamine induce passive avoidance test [136]. A few years later, the same research group published new urolithin-based MTDL compounds, obtained from combination of urolithin or tetrahydrourolithin with donepezil-like scaffolds throughout a propylene linker (20, Table 4). Even if these compounds exhibited good anticholinergic activity, they lack the structural requirements to prevent amyloid beta aggregation inhibition, suggesting that the design of new AChEI should not be sufficient to prevent cholinesterase induced Aβ aggregation [137].

Recently, the modulation of the casein kinase system (CK) has emerged as new therapeutic approach for NDDs. In particular, CK2 is a ubiquitous protein kinase that seems to play important roles in neural functions including synaptic transmission, and synaptic plasticity, suggesting a potential critical role also in the progression of AD [138]. Cozza et al. proposed urolithins scaffold as a starting point for designing new and promising CK2 inhibitors. New urolithin derivatives (21, Table 4) showed highly selective sub-micromolar activity against CK2, suggesting that they could led the basis of new approaches against NDDs [139,140]. In 2016, Xie et al. proposed new donepezil-coumarin multi-target hybrids, identifying tetrahydrourolithin derivatives as potential therapeutic tools for AD (22, Table 4). Indeed, they showed good in vitro inhibition of *h*AChE and *h*BuChE (IC_50_ = 1.37 μM and 1.98 μM, respectively), and MAO-B (IC_50_ = 2.62 μM), and no inhibition towards MAO-A. Moreover, tetrahydrourolithin derivatives showed an interesting in vitro capacity of permeating the BBB, and no cytotoxicity in SH-5YSY and HepG2 cells at concentrations up to 50 μM [141].

### 3.4. Lipoic Acid

**Figure 4:**
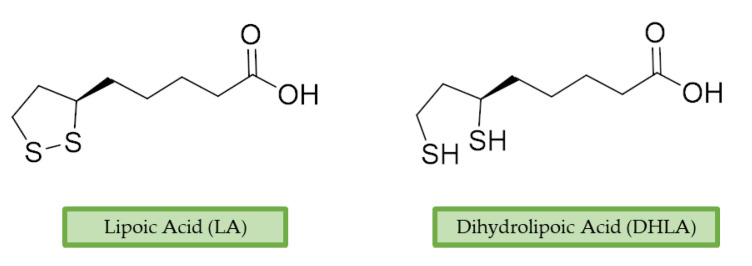
Lipoic acid (LA) and its reduced form, dihydrolipoic acid (DHLA).

α-Lipoic acid (LA) is a natural disulfide antioxidant compound occurring in vegetables, meat, and fruits. In cells LA is converted to dihydrolipoic acid (DHLA) and plays a prominent role as co-factor for mitochondrial dehydrogenases—i.e., pyruvate dehydrogenase (PDH) and α-ketoglutarate dehydrogenase (KGDH) [142]. Since the 1950s, antioxidant properties of LA have been investigated, identifying in this compound a beneficial micronutrient that could find potential therapeutic application against OS and NDDs [143] (Figure 4).

In vitro evidence observed a direct radical scavenging capacity of LA against OS [144]. However, recent in vivo studies have suggested that LA and DHLA effects are mainly due to the improvement of antioxidant enzymes levels—such as CAT, SOD, GPx and glucose-6-phosphate dehydrogenase (G6PD) [145]. LA restored age-declined GSH levels in rats CNS, sustaining antioxidant defences and protecting against aging alterations [146]. Retention of GSH basal levels and promotion of antioxidant effects were also observed in subarachnoid hemorrhage rat model after LA administration [147]. Additionally, LA is capable of chelating transitional metals, taking part in prooxidant Fenton reactions, and is involved in protein aggregation. Studies employing in vitro model of iron-overload and dietary administration of LA in rats indicated that LA possess antioxidant and neuroprotective effects associated with its iron-chelating properties [148]. Subsequent studies reported good chelating properties both in vitro and in vivo models against additional metals including manganese, aluminium [149,150], and copper [151,152]. In 2019, Bjørklund et al. reviewed the protective chelating properties of LA and other thiol groups containing compounds, towards mercury, cadmium, and lead accumulation [153]. LA has also shown beneficial effects related to inhibition of lipid peroxidation: in patients affected by diabetic neuropathy LA showed a neuroprotective action reducing oxidative stress and lipid peroxidation [154]. In two separate experiments on rats, LA showed antioxidant activity, BBB protection, and a remarkable decrease of lipid peroxidation [147,155]. 

Since 2000, LA effects on cholinergic system have been deeply studied [156]. LA ameliorated cholinergic deficiency in vascular dementia rats model: levels of ACh and choline acetyltransferase (ChAT) were partially restored along with a decreased AChE activity. Chronic dietary intake of LA in aged Tg2576 mice reduced cognitive deficits induced by amyloid precursors overexpression, recovering spatial learning and memory retention without affecting brain Aβ levels [157]. Due to the multiple activities and the good pharmacokinetics, LA is currently the most studied nature-based compound in clinical trials and suggested as a new treatment option for NDDs including AD, ALS, and multiple sclerosis [158,159]. Based on these promising results, LA has been widely investigated as lead-structure for designing MTDL compounds. One of the first LA-based anti-AD multi-target compound was proposed by Rosini et al. as a combination of LA with the tacrine scaffold (23, Table 5). Lipocrine showed in vitro inhibition of AChE, BuChE, AChE-induced Aβ aggregation and cells protection against ROS [160]. Through the years, the same research group designed and evaluated other LA-multi-target analogues containing substituted tacrine, memoquin, and rivastigmine moieties with promising potential in OS and protein aggregation treatments [161]. Similarly, Table 5 summarized some examples of LA-multitarget ligands designed as neuroprotective and antioxidant molecules for the treatment of AD, thus proving its promising properties for the treatment and/or prevention of neurodegenerative disorders. For each LA-derivative, main pharmacological effects and pharmacophoric portions are indicated: dopamine (24), AChE inhibitors (25), hydrophobic portion of SR3677, a potent Rho-associated kinase 2 (ROCK2) and Aβ inhibitor (26), isosorbide (27), coumarin (28), phenolic acids (29), benzodiazepine (30), melatonin (31), niacin (32), and 3-n-butylphthalide (NBP), an anti-ischemic drug (33) [162,163,164,165,166,167,168,169,170,171].

## 4. Discussion

In the last 50 years, interest in bioactive natural products has increased considerably. Indeed, extensive studies have been conducted on well-known substances (e.g., polyphenols and phenolic acids), identifying a large number of previously unknown therapeutical effects. Additionally, the impressive advancements in extractive techniques and mass spectrometry allowed the isolation and identification of several previously undetectable active compounds (e.g., urolithins). In parallel, our knowledge on complex uncurable pathologies has widely progressed towards the identification of several mechanisms (e.g., autophagy, proteostasis) involved in their pathogenesis, leading to a paradigm-shift in our conception of several diseases. Beside neoplastic diseases, NDDs have probably received most of the attention of pharmaceutical researchers and drug discovery programs. The identification of a vast tangled network of pathogenic mechanisms responsible of the onset and progression of NDDs has forced the academic world and pharmaceutical industries to re-think the therapeutic approaches to pursue. As discussed above, excessive OS correlates well with several dysfunctionalities observed in these pathologies resulting an intriguing target for new pharmacological agents. However, the development of new drugs able to promote a rebalance of OS still represents a difficult goal to achieve, especially since physiological levels of OS mediators (e.g., ROS) are required for maintaining healthy conditions.

In this context, polypharmacology associated with natural products have gained a prominent role in the drug discovery process for NDDs; intriguingly, the first drug approved by the Chinese regulatory authority in 2019 for AD is a gut-microbiota reprograming agent identified in seaweeds [172]. However, most NDDs (e.g., AD, PD, ALS) still lack a proper efficacious therapy able to halt, reduce or reverse their pathological progression. Interestingly, last years have seen an exceptional increase in clinical trials employing dietary enrichment with natural products (e.g., spermidine) or administration of natural extracts in NDDs patients [73,173]. Particularly, one of the most successful and promising cases is represented by ECGG that is currently in phase 2 as dietary supplement for AD and phase 3 for ALS (source: www.alzforum.org) (accessed on 11 February 2021). However, most of bioactive natural small molecules lack fundamental features required for being successfully delivered as NDDs drugs. Low aqueous solubility, poor PK and PD profiles, as well as high metabolic in vivo conversion, strongly hinder the progress of most natural products through the drug discovery chain. Accordingly, the MTDL approach could help to perfectly merge polypharmacology and natural products benefits, generating new chemical entities with the pharmacological fingerprints of natural products and able to simultaneously target two or more pathological mediators.

## 5. Conclusions

In the last years, several attempts have been made towards the development of new naturally derived polypharmacological agents and many new multitarget drugs with improved pharmacological features and optimized PK/PD profile have been synthesized. In this review, we summarized a large number of nature-based multitarget drugs developed in the last 10 years. Particularly, we focused on polyphenols and polyamines that share a prominent antioxidant effect. This feature could play a key role in developing new therapies for multifactorial diseases like NDDs, but not only. Indeed, we widely discussed how their therapeutical potential goes beyond the known antioxidant effects, playing a key role in defending the organism from excessive OS and having demonstrated a plethora of beneficial effects, including decreasing Aβ aggregation, promoting autophagy, and reducing metal accumulation. However, few issues have yet to be addressed. To the best of our knowledge, none of the nature-based multitarget molecules presented in this review has progressed through the drug discovery chain to successfully reach the market. The promising activity showed in vitro and even in preclinical studies still required to be validated in human subjects affected by NDDs. Nevertheless, the overview provided by the polypharmacology associated with natural compounds—and their synthetic derivatives—suggests that nature-based templates could represent a valuable tool for the drug design in the effort to realize new valuable drugs for NDDs.

## Figures and Tables

**Table 1:** Main physiological and pathological roles of ROS.

Physiological Role of ROS	Ref.	Pathological Role of ROS	Ref.
Signalling between mitochondria and surrounding cells	[20]	mDNA damage, deletion, and mutation	[21]
Regulation of cellular proliferation, differentiation and apoptosis	[18,19]	Mitochondrial membrane permeability alteration and mitochondrial failure	[22,25,27,31]
Induction of MAPKs activation in cardiovascular system.	[20]	Lipid peroxidation	[27]
Influence on pro-survival transcription factors (i.g. Nrf2 and NF-κB).	[21]	ETC enzymes malfunctions	[29]
Adaption and regulation of hypoxia	[20]	Promotion of inflammation	[16]
Regulation of immune functions	[19,20]	Metallostasis and metal accumulation	[40,42]
Induction of autophagy	[20]	Proteostasis and misfolded proteins clearance impairment	[51,53]

**Table 2:** Polyamines derivatives and their main biological effects.

Entry	Scaffolds Combination	Structure	Effects	Ref.
1	1-aminoindan beared with polyamine scaffold	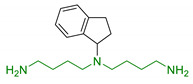	Neuroprotection against NMDA toxicity and ischemia damages No neurotoxicity	[85]
2	1,4-benzoquinone and polyamine structure of caproctamine	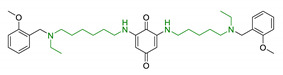	↓Aβ aggregation ↓tau phosphorylation ↑antioxidant activity ↓AChE ↓BACE-1	[87]
3	Ferulic acid-memoquin hybrids	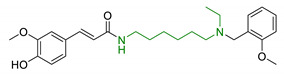	↓AChE ↓BuChE ↓self-induced Aβ_1-42_ aggregation no cytotoxicity in SH-SY5Y cells good BBB predicted permeability	[88]
4	Genistein with polyamines	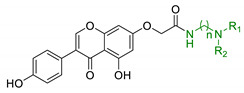	↓AChE ↓BuChE Fe^3+^/Cu^2+^/Zn^2+^ chelation no HepG-2 cell cytotoxicity	[89]
5	3,5-dibenzylidenepiperidin-4- one functionalized with spermine	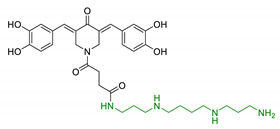	↓Aβ_42_ aggregation no antioxidant properties in T67 cells neuroprotection and no cytoxicity in vitro	[90]
6	Dicaffeoylsper-midine cyclized derivatives	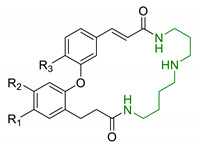	Antioxidant activity ↑memory and learning in fruit flies model	[92]

**Table 3:** Phenolic acids hybrids and their main biological effects.

Entry	Scaffolds Combination	Structure	Effects	Ref.
7	Tacrine linked with ferulic acid	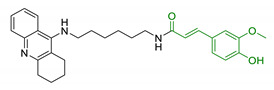	↓ Aβ-aggregation ↓ ROS production ↓ AChE ↑ cognitive functions ↑ SOD/ChAT	[110]
8	Tacrine and functionalized ferulic acid	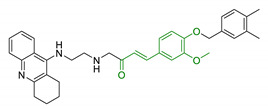	↓ Aβ-self aggregation ↓ AChE ↓ BuChE ↑ memory no hepatotoxicity	[111]
9	Ferulic and caffeic merged with serotonin	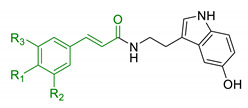	↑ antioxidant activity ↓ BACE-1	[112]
10	Aromatic amides and esters of caffeic acid	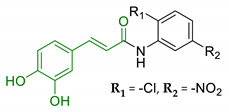	↓MAO-A/MAO-B ↑ antioxidant activity	[113]
11	Hydroxycinnamic acids and NBP (donepezil)	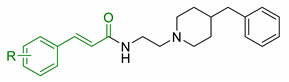	↓MAO-A/MAO-B ↓AChE ↓BuChE ↑ antioxidant activity	[114]
12	Hydroxycinnamic scaffolds and DBMA (AP2238)	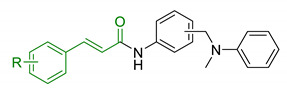	↓MAO-A/MAO-B ↓AChE ↓BuChE ↑ antioxidant activity	[114]
13	Caffeic acid with hydrophobic moieties	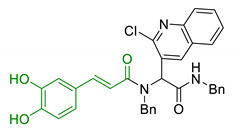	↓Aβ_1-40_ self-aggregation ↑ antioxidant activity neuroprotection in SH-SY5Y cells	[115]
14	Rivastigmine with GA	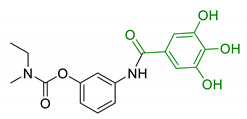	↑ antioxidant activity Cu^2+^ chelating properties ↓ChEs ↓Aβ self-aggregation neuroprotective effects in vitro no cytotoxicity	[116]
15	Caffeic acid and diallyl sulfide	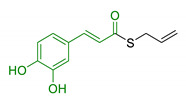	↓Aβ_42_ self-aggregation ↑cytoprotection against H_2_O_2_-induced damages ↓p53 alteration induced by Aβ	[117]
16	Ferulic core merged with 1,2,3,4-tetrahydroisoquinoline and (benzyl(ethyl)amino)butoxy scaffold	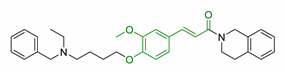	↑antioxidant activity ↓AChE ↓BuChE ↓ MAO-A/MAO-B ↓ Aβ self-aggregation ↑ self-induced Aβ_1-42_ fibrils disaggregation ↑neuroprotective effect in SH-5YSY cells ↑autophagy in U87 cells ↑motility in Zebrafish model ↓ Aβ_1-40_-induced vascular injury in Zebrafish model ↑In vivo cognitive functions	[118]
17	Ferulic acid merged with 1,3,4-oxadiazole scaffold	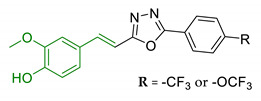	↓ChEs ↓BACE-1 ↓ Aβ self-aggregation ↓ Aβ AChE-induced aggregation neuroprotective effects in vitro ↑In vivo cognitive functions	[119]

**Table 4:** Urolithins derivatives and their main biological effects.

Entry	Scaffolds Combination	Structure	Effects	Ref.
18	Urolithin scaffold with rivastigmine portion	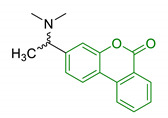	↓AChE ↓BuChE	[136]
19	Urolithin scaffold with donepezil-like moieties	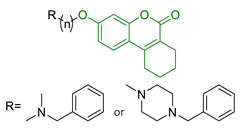	↓AChE ↓BuChE	[136]
20	Donepezil-like urolithin and tetrahydrourolithin derivatives	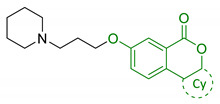	↓AChE ↓BuChE ↓ AChE induced Aβ aggregation	[137]
21	Nitro- and bromo-derivatives of urolithins	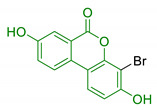	CK2 inhibition Selectivity in other kinases panel	[140]
22	Tetrahydrourolithin scaffold linked with donepezil moiety	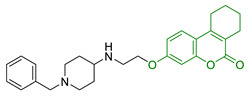	AChE/BuChE inhibition MAO-B inhibition BBB permeability no cytotoxicity in brain and liver cells	[141]

**Table 5:** Lipoic acid hybrids and their main biological effects.

Entry	Scaffold Combination	Structure	Effects	Ref.
23	Lipoic acid and tacrine	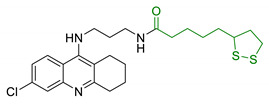	↑ROS protection ↓AChE ↓BuChE ↓ AChE-induced Aβ aggregation	[160]
24	Dopamine and LA linked by tetrazole ring	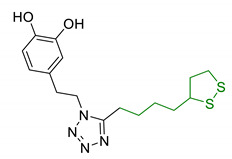	↑antioxidant activity neuroprotection in vitro	[162]
25	LA-NBP and LA-DBMA conjugation	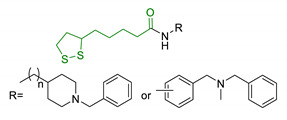	↓AChE ↓BuChE ↓BACE-1 ↑antioxidant activity σ1R agonism good BBB permeability prediction neuroprotection in vitro	[163]
26	LA-4-Phenyl-1H-pyrazole derivatives	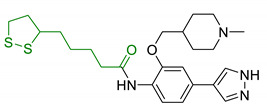	ROCK1/ROCK2 inhibition ↓ROS ↑GSH vasorelaxant activity	[164]
27	Lipoic isosorbide-2-benzylcarbamate	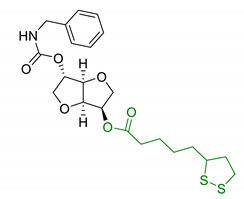	↓ROS ↓BuChE ↓cytotoxicity in treated HT-22 cells	[165]
28	LA and coumarin scaffold linked bridged with triazole	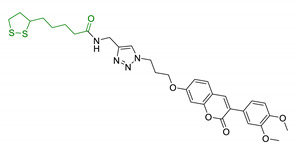	↓AChE ↓BuChE ↓ Aβ peptide aggregation ↓intracellular ROS neuroprotection against H_2_O_2_− or Aβ_1-42_-induced cytotoxicity in SH-SY5Y cell lines Selective Cu/Fe chelation	[166]
29	FA/CA-LA hybrids	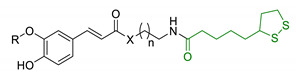	↓Aβ_1-42_-induced neurotoxicity in SH-SY5Y cells ↑protection in H_2_O_2_-insulted cells no cytotoxicity	[167]
30	Lipoic-functionalized benzodiazepine	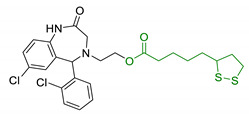	↑ROS scavenging ↑Nrf2-ARE pathway ↑HO-1/GCLc neuroprotection in in vitro model no cytotoxicity no hepatotoxicity	[168]
31	Lipoic-melatonin hybrids	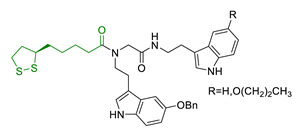	↑ROS scavenging ↑Nrf2-ARE pathway antioxidant activity and neuroprotection in vitro no cytotoxicity	[169]
32	LA-niacin hybrids	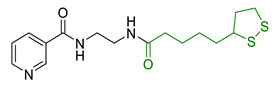	↓Aβ_1-42_-induced cytotoxicity in HT22 cells ↓mitochondrial dysfunctions ↓intracellular ROS ↑SOD, CAT, GPx ↓apoptosis in Aβ_1-42_treated cells	[170]
33	LA-3-n-butylphthalide amide	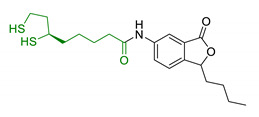	↓intracellular ROS ↑direct ROS-scavenger ↓ H_2_O_2_-induced cell death ↑GSH ↓ H_2_O_2_-induced damage in cortical neurons ↓6-OHDA-induced neuronal damage in SH-5YSY cells	[171]
